# Strategies for Enhancing *in vitro* Degradation of Linuron by *Variovorax* sp. Strain SRS 16 Under the Guidance of Metabolic Modeling

**DOI:** 10.3389/fbioe.2021.602464

**Published:** 2021-04-15

**Authors:** Kusum Dhakar, Raphy Zarecki, Daniella van Bommel, Nadav Knossow, Shlomit Medina, Basak Öztürk, Radi Aly, Hanan Eizenberg, Zeev Ronen, Shiri Freilich

**Affiliations:** ^1^Newe Ya’ar Research Center, Agricultural Research Organization, Ramat Yishai, Israel; ^2^Department of Environmental Hydrology & Microbiology, Zuckerberg Institute for Water Research, Jacob Blaustein Institutes for Desert Research, Ben-Gurion University of the Negev, Beersheba, Israel; ^3^lbert Katz School for Desert Studies Jacob Blaustein Institutes for Desert Research, Ben-Gurion University of the Negev, Beersheba, Israel; ^4^Junior Research Group Microbial Biotechnology, Leibniz Institute DSMZ, German Collection of Microorganisms and Cell Cultures, Braunschweig, Germany

**Keywords:** phenyl urea herbicide, linuron, 3, 4-dichloroaniline, biodegradation, genome-scale metabolic model, *Variovorax* sp. strain SRS 16

## Abstract

Phenyl urea herbicides are being extensively used for weed control in both agricultural and non-agricultural applications. Linuron is one of the key herbicides in this family and is in wide use. Like other phenyl urea herbicides, it is known to have toxic effects as a result of its persistence in the environment. The natural removal of linuron from the environment is mainly carried through microbial biodegradation. Some microorganisms have been reported to mineralize linuron completely and utilize it as a carbon and nitrogen source. *Variovorax* sp. strain SRS 16 is one of the known efficient degraders with a recently sequenced genome. The genomic data provide an opportunity to use a genome-scale model for improving biodegradation. The aim of our study is the construction of a genome-scale metabolic model following automatic and manual protocols and its application for improving its metabolic potential through iterative simulations. Applying flux balance analysis (FBA), growth and degradation performances of SRS 16 in different media considering the influence of selected supplements (potential carbon and nitrogen sources) were simulated. Outcomes are predictions for the suitable media modification, allowing faster degradation of linuron by SRS 16. Seven metabolites were selected for *in vitro* validation of the predictions through laboratory experiments confirming the degradation-promoting effect of specific amino acids (glutamine and asparagine) on linuron degradation and SRS 16 growth. Overall, simulations are shown to be efficient in predicting the degradation potential of SRS 16 in the presence of specific supplements. The generated information contributes to the understanding of the biochemistry of linuron degradation and can be further utilized for the development of new cleanup solutions without any genetic manipulation.

## Introduction

Phenyl urea herbicides are among the most widely used herbicides for weed control in several crops (mostly cereals) through their pre- or post-emergence applications. These substances interrupt electron transfer in photosystem II, leading to the formation of reactive oxygen species and resulting in cell damage (Liu, 2010). The increased rate of application of xenobiotics such as the phenyl urea herbicides in recent years enhanced their burden to the environment due to their persistence in the surroundings (Hasanuzzaman et al., 2020). These compounds are consistently found to have negative effects on the ecosystem, including hazards to human health (de Souza et al., 2020; Garcês et al., 2020). Linuron has been one of the most widely applied phenyl urea herbicides in agriculture practice, also reported as an environmental pollutant (Dejonghe et al., 2003; Horemans et al., 2016). Remediating the environment from the accumulated toxic substances is the focus of a growing number of researches efforts. The degradation or the removal of such pollutants is reported to be conducted through physical, (photo-)chemical, and chemical processes (Katsumata et al., 2011; Reddy and Kim, 2015; Kovács et al., 2016; Hao et al., 2019; Bhat et al., 2020). Microorganisms play a major role in the biological removal of linuron. So far, different strategies have been approached for enhancing biodegradation and bioremediation (Raman and Chandra, 2009; Pimviriyakul et al., 2020). By promoting the growth of soil microbial degraders, soil amendments are being used in order to accelerate the removal rate of pollutants (Bao et al., 2020). However, the selection of the amendments is mostly based on trial and error.

Biodegradation of linuron is generally initiated by amidase hydrolases, which leads to the formation of a more toxic intermediate, 3,4-dichloroaniline (DCA). DCA further degrades to metabolites that can be consumed in the central metabolism of the microorganisms. However, partial degradation of linuron produces chloroanilines that are more toxic than linuron itself. Bacterial genera such as *Arthrobacter*, *Bacillus*, *Comamonas*, *Pseudomonas*, *Sphingobium*, and *Variovorax* are able to degrade/transform linuron in various environments either in isolation or as part of a consortium (Turnbull et al., 2001; Dejonghe et al., 2003; Lerner et al., 2020; Zhang et al., 2020). *Variovorax* SRS 16 is widely studied for its ability to utilize linuron as a sole carbon and nitrogen source. *Variovorax* SRS 16 also possesses a modified chlorocatechol ortho-cleavage pathway, allowing the utilization of linuron as a substrate for growth. The presence of the amidase (specific linuron hydrolase) and gene clusters which are responsible for the degradation of linuron and dichloroaniline in SRS 16 has been described in detail with the support of proteomic studies (Bers et al., 2011). Based on the relatively extensive phenotypic information, together with the publication of its genome sequence (Sørensen et al., 2005; Bers et al., 2011), *Variovorax* SRS 16 is recognized as a good model for exploring biodegradation of phenyl urea herbicides.

Processing genomic information into a metabolic model is increasingly used as a route for generating a predictive tool to elucidate and manipulate cellular biochemical activity. Genome-scale metabolic modeling has been proven as an efficient approach to decode the genomic and functional information for a specific phenotype by investigating the gene–protein interactions on a cellular level (Thiele and Palsson, 2010; Gu et al., 2019). To construct metabolic models, some preliminary information on physiological requirements (mainly of growth) of the organism is needed for better curation and to ensure the appropriate functioning of the model (Covert et al., 2001). This approach generally follows some defined steps such as collecting basic information related to the conditions required for growth of cells together with description of the metabolism of the organism based on its genome sequence. Conversion of this information into a mathematical framework, as a model, is the next step. Further, the behavior of the model can be predicted under specific growth conditions along with biomass generation and exchange (uptake or release) of relevant compounds (metabolites). In the final step, experiments are carried out to validate the predictions (Devoid et al., 2013). Genome-scale modeling is considered advantageous as it allows screening of multiple conditions in a short time and lowering of cost by providing a limited set of solutions that can be further tested. Solutions that enhance a desired or improved behavior of the organism can be predicted based on media supplementations without any requirement of genetic modification (García-Jiménez et al., 2018).

The importance of these models as a tool for rapid biodegradation and for the development of effective bioremediation strategies is recognized (Scheibe et al., 2009) and demonstrated in a few studies. For example, a metabolic model of *Ralstonia eutropha* H16, a microorganism with multiple potential applications in environmental biotechnology, was used to analyze its metabolic characteristics (Park et al., 2011). The genome-scale model of *Pseudomonas veronii* 1YdBTEX2, a degrader of monoaromatic compounds, was used together with ‘omics data (transcriptomics and exometabolomics) to characterize active metabolic processes at different growth stages. The matching of predictions with bacterial response indicates the importance of modeling to enhance the success rate of bio-inoculants in complex systems (Hadadi et al., 2020). Such studies help to predict the changes in cellular processes under a specific given condition (e.g., xenobiotic biodegradation). In addition, such integrative “omics modeling” approaches have also been used for optimization of predefined processes (Calmels et al., 2019). Xu et al. (2019) used models constructed for indigenous species whose abundance in soil was affected by exposure to the herbicide atrazine. The community modeling revealed interspecies metabolic interactions that support enhanced growth and degradation. The simulations pointed at a contribution of non-degrader species to the *in situ* process achieved thorough trophic exchanges with the degrader (Faust, 2019). The effect of specific metabolites on the degradation rate of atrazine by *Paenarthrobacter aurescens* TC1 was predicted by a genome-scale model and was further validated *in vitro* (Ofaim et al., 2020). The predictive biology facilitated the identification of optimal conditions for degradation that can promote the development of bioaugmentation or biostimulation approaches for better controlling the degradation processes. One bottleneck of bioaugmentation is that in many cases, the exogenous degrading microorganisms fail to establish in a new environment and do not produce enough biomass to eliminate contaminants *in situ*. Stimulating the growth of native degraders through adding specific compounds to the contaminated site is a practical alternative to bioaugmentation. The present study aims to elucidate the effect of nutritional supplements on linuron degradation by *Variovorax* SRS 16 (as a model for native degraders) and estimate their potential usage as biostimulants in order to harness the full potential of indigenous degraders. SRS 16 was selected due to the availability of a fully sequenced genome and extensive biochemical knowledge of its full pathway in linuron degradation – a pollutant that belongs to a large contaminant group of the phenyl urea herbicides. A genome-scale metabolic model of SRS 16 was constructed following automatic and manual protocols and was applied to explore the stimulating effect of different supplements through iterative simulations Compounds pointed by simulations as strong enhancers of degradation were further validated through wet laboratory experiments.

## Materials and Methods

### Constraint-Based Reconstruction

As a first step, a draft model of *Variovorax* sp. SRS 16 was reconstructed by following a bottom-up approach as elaborated by Ofaim et al. (2020). Briefly, the whole-genome sequence of SRS 16 was retrieved from the genome database of NCBI and annotated using RAST (Overbeek et al., 2014). An initial metabolic reconstruction was obtained by analyzing the annotated genome sequence through Model SEED (Faria et al., 2018). The metabolic reconstruction has three main components: a complete list of reactions and their associated genes, information of interactions between the gene and protein associated with the metabolic reaction, and the components involved in biomass generation (Devoid et al., 2013). The list of reactions contains the biomass (which includes all the biomass constituents and their fractional status), cytosolic, transport, and exchange reactions. The model was improved by adding potentially missing reactions through an automated gap-filling process (Henry et al., 2010). The improved draft model was exported to Systems Biology Markup Language (SBML) format from Model SEED.

**FIGURE 1:**
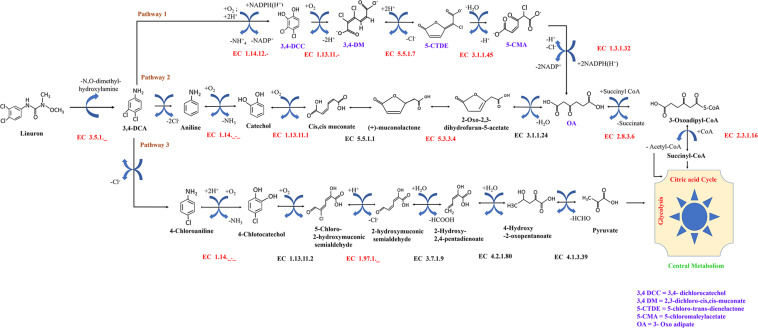
Possible fate of linuron and 3,4-DCA in SRS 16. Reactions in black were included in the automatic reconstruction. Reactions in red were manually added to the model. Pathway 1 (modified ortho-cleavage pathway) was retrieved from BioCyc based on direct evidence for *Variovorax* SRS 16 strain (https://biocyc.org/META/NEW-IMAGE?type=PATHWAY&object=PWY-7496). Pathways 2 and 3 (ortho- and meta-cleavage pathways, respectively) were completed by following the generic KEGG pathways.

The reactions in the original model were complemented by reactions described for *Variovorax* SRS 16 in additional genomic annotation databases including BiGG (Norsigian et al., 2020), KEGG (Kanehisa et al., 2016), UniProt (The UniProt Consortium, 2019), IMG (Chen et al., 2019), and MetaCyc (Karp, 2002). Reactions were included according to EC accessions. Reaction stoichiometries were verified for all reactions, and non-balanced reactions were fixed. Directionality and stoichiometry of reactions were determined according to the KEGG scheme. Specific reactions involved in linuron degradation were added based on a literature survey (Figure 1). The reconstruction was curated to verify that it allows the generation of all biomass components under physiologically feasible minimal conditions – minimal salt solution and linuron (Supplementary File 1). The *Variovorax* sp. SRS 16 model in SBML format is provided in Supplementary File 2.

### Simulations of Growth and Degradation of Linuron Using Flux Balance Analysis

Simulations were carried out using flux balance analysis (FBA), allowing us to depict cellular processes based on cellular reconstructions (Rana et al., 2020). Briefly, FBA follows the law of mass conservation and consider the metabolic framework as a static stoichiometric matrix (metabolites × reactions). FBA describes the predicted flux distribution through linear optimization by targeting specific cellular objectives (mainly growth). In the present study, the growth of the model was chosen as the objective function through the maximization of the biomass reaction. Flux variability analysis (FVA) was carried out to account for the possible flow of fluxes involved in secretion and uptake of all metabolites (Mahadevan and Schilling, 2003). The simulations were applied to predict biomass generation and linuron degradation over a time period under a range of defined conditions – *in silico* growth media. All simulations were carried out under definitions that follow experimentally verified viable conditions in minimal media with linuron (Supplementary File 3) and 120 exchange metabolites (one at a time) representing an alternative carbon/nitrogen source or other supplements (Supplementary File 4).

Dynamic modeling was used for the prediction of the profile of consuming metabolites typical to the biomass increase and linuron degradation across time. To this end, we simulated the behavior of our metabolic model across time. The simulation process follows (Xu et al., 2019; Ofaim et al., 2020) and is illustrated in Supplementary File 5. Briefly, the model works under the following assumptions: (1) a finite start amount of media components is available; (2) a maximal amount of uptake a single cell can acquire from the media in a given time point is defined (the lower bound of the exchange reaction value); (3) new substrate concentrations in each time point are determined by the predicted substrate concentration for the previous step augmented with any additional substrates provided or consumed in the current iteration. The maximum uptake was set to a ratio of up to 1 unit of each metabolite available in the media; (4) after each time tick, the biomass amount was updated according to the flux amount of the biomass reaction in the model at this time tick. As the biomass production rate serves as a proxy for the size of the population in the simulated environment and substrate uptake/secretion is mainly affected by population size, the model was used to evaluate the actual substrate uptake and growth rate given the supplied media across time.

Simulations were carried out until a state where time cycles did not lead to an increase in biomass was reached. Initial concentration values for all metabolites were set to a fixed amount of 50 units (represented as the initial lower bounds, LB, of the exchange reactions). The upper bound of the exchange reactions was set to 1,000 units to allow the secretion of all the exchange metabolites. Reversible non-exchange reactions’ lower bound was −1,000 units, and for non-exchange non-reversible reactions, it is 0 units; the upper bound for non-exchange reactions was 1,000 units. At each growth cycle, the generation of biomass is updated on the basis of flux flow in the biomass reaction. The algorithm assumes that media components are available to all growing cells with equal probability. The growth rate and the flux of substrate (consumed or secreted) associated with the model in the given media conditions are calculated at every time point, where, in each cycle, we try to maximize the biomass of each member of the community and then we fixate the biomass and minimize the uptake of metabolites of each member while maintaining the max biomass found. The cycles of predictions were carried out until the saturation point appeared in the biomass production (no growth recorded reflecting the exhaustion of given resources). Starting with one bacterial cell, the flux balance model was used to predict the uptake of carbon and nitrogen sources (including linuron) across time.

All model simulations were done on an Intel i7 quad-core server with 32 GB of memory, running Linux. The development programming language of our simulators was JAVA, and our linear programming software was IBM CPLEX.

### *In vitro* Experiments of *Variovorax* sp. SRS 16 Strain: Growth and Linuron Degradation

*Variovorax* sp. strain SRS 16 (NCBI: txid282217, kindly provided by Dirk Springael, KU Leuven) was revived from stock cultures stored in glycerol at −80°C. The purity and authenticity of the strain were checked by 16S rRNA gene sequencing. The minimal medium (Supplementary File 3) described by Sørensen and Aamand (2003) was prepared, and bacterial cells were grown on agar plates at 25°C. For the quantitative analysis, 250 ml flasks were used to prepare the minimal medium (50 ml of the medium per flask) and autoclaved. The effects of the seven selected substrates – representing strong, moderate, and weak enhancers of linuron degradation – on growth and linuron degradation were tested. The supplements (filter sterilized) were added to a final concentration of 0.12 mM in media (equivalent to 30 ppm of linuron). The linuron concentration was selected on the basis of a previous study. MS, MS + C, and MS + N consist of only minimal medium, minimal media added with only carbon (glucose), and minimal media added with only nitrogen (ammonium salt), respectively. MS was treated as a negative control. The autoclaved minimal medium was supplemented with the substrates (separately) and linuron (30 ppm) followed by bacterial inoculation to a final OD (at 600 nm) of 0.05–0.1. The mother culture was raised in MSCN (succinic acid and ammonium salt) medium with linuron, by inoculating fresh agar plate-grown bacterial cells followed by incubation at 25°C (120 rpm) for 24 h.

All the inoculated flasks were incubated at 25°C (120 rpm) for 7 days. Bacterial growth and linuron degradation were monitored at definite intervals (zeroth day, third day, fifth day, and seventh day) from the day of inoculation. For bacterial growth, 200 μl was taken, and OD was measured at 600 nm by using Infinite^®^ 200 PRO (Tecan Trading AG, Switzerland). Linuron degradation was measured through HPLC with standard procedures. Briefly, 1 ml of sample was taken and centrifuged at 10,000 × *g* for 5 min. The supernatant was filtered through a 0.22 μm PTFE syringe filter and transferred to the HPLC vials for the detection of residual linuron. Linuron and DCA were analyzed by using Agilent 1100 HPLC (Waldbronn, Germany). Detection of linuron was done at 240 nm using the external calibration method (sensitivity 0.5 mg L^–1^). The mobile phase of 70% methanol at a flow rate of 1 ml min^–1^ with a reverse-phased (Phenomenex, Torrance, CA) of 250 mm length × 4.6 mm inner diameter with particle size 5 μm was used for separation.

Linuron biodegradation and biomass buildup experiments were carried out in biological triplicates. The effect of supplements was statistically analyzed by performing repeated-measures ANOVA at *p* < 0.05 in SPSS v19.

## Results

### Reconstruction of Genome-Scale Metabolic Network for *Variovorax* sp. Strain SRS 16

The genome of *Variovorax* sp. strain SRS 16 is about 7.7 Mb in size, including its chromosome (5.7 Mb) and four plasmids with an approximate size of <1 Mb each (Öztürk et al., 2020). The complete sequence was retrieved from NCBI and annotated for reconstruction using the Model SEED pipeline. This initial reconstruction contained a list of 2,150 gene–protein*–*reaction associations that were classified as exchange, transport, and cytosolic as well as a list of all relevant metabolites and a biomass reaction. Based on the taxonomic classification, the biomass reaction was defined as Gram-negative bacteria in the Model SEED reconstruction pipeline. The composition of the biomass reaction summarizes the fractional contribution of generalized microbial biomass precursors (e.g., amino acids and lipids) to the synthesis of a new cell and is similar to the previously published genome-scale reconstruction of *Escherichia coli* strain K-12 (Monk et al., 2017). Initial simulations were carried out for debugging and removing futile or erroneously energy-generating loops. To this end, all external fluxes were blocked (upper and lower bounds set to zero). Next, a minimal medium was used, verifying that growth requires the supply of both carbon and nitrogen sources. After establishing no growth under infeasible conditions, we tested growth (biomass production) under experimentally verified conditions (succinic acid and ammonium salt as a carbon and nitrogen source, respectively) as described by Sørensen et al. (2009). Fine tuning of growth simulations to correctly represent the bacteria’s biology was done by manual curation. This included manual gap filling (addition of spontaneous and literature-supported reactions) and curation of reaction directionality.

The reconstructed metabolic network presented here covers 19.2% of the open reading frames (ORFs) present in the genome (Table 1). The coverage is lower than gold standard models of other Gram-negative models such as iAF1260 for *Escherichia* (Feist et al., 2010) (29%), iPC1209 for *Pectobacterium* (Wang et al., 2015), and iPAO1 for *Pseudomonas* (25.8%) (Zhu et al., 2018). It is, however, similar to coverage obtained for the genome-scale reconstruction of the closely related genus *Rhodoferax ferrireducens* (from the same family *Comamonadaceae*) – 15.6% (Risso et al., 2009). Total number of reactions is similar to gold standard reconstruction including the updated model of *E. coli* str. K-12 (Orth et al., 2011). A detailed description of the network including the reactions, metabolites, genes, and compartments that comprise the network is provided in Table 1. The model is also available as an SBML file (Hucka et al., 2003) in Supplementary File 2. The SBML file can be used with tools such as MATLAB or other SBML-compliant software. The minimal media used for simulations are available in Supplementary File 1. The set of metabolites, reactions, and exchanges are provided in Supplementary Files 1, 4.

**TABLE 1:** Functional details of the *Variovorax* sp. SRS 16 model.

Serial no.	Category	Total number
1	Metabolites	2,185
2	Exchange reactions	127
3	Reversible reactions	1,432
4	Transport reactions	277
5	Biochemical reactions	1,746
6	Total reactions	2,150
7	Genes associated with a reaction*	1,425 (total 7,411) (Öztürk et al., 2020)

*19.2% coverage of genome.

### Adapting and Curating iRZ1425 for Modeling Linuron Degradation

**FIGURE 2:**
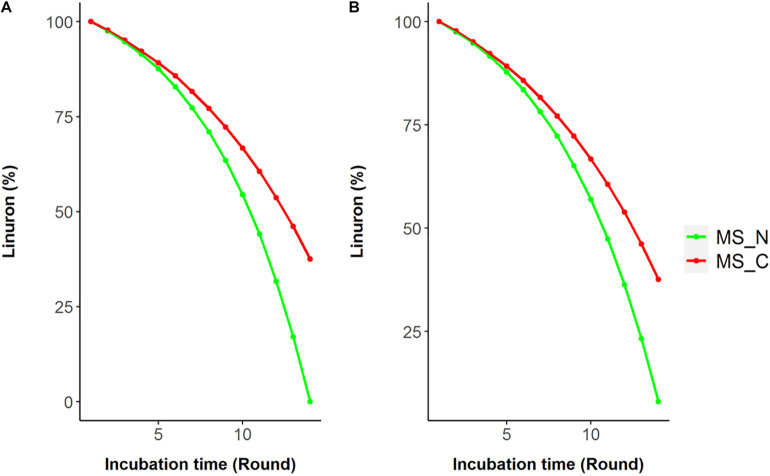
Simulated linuron degradation in the modified media SRS 16 model considering Pathway 1 **(A)** and Pathways 2 and 3 **(B)**. Simulations were carried out in minimal mineral media (MS) with linuron supplemented by nitrogen (ammonium sulfate) or carbon (succinate) sources (MS_N and MS_C, respectively).

Model reactions involved in linuron degradation and their complete link to the core metabolism were added manually based on the detailed reports of the relevant pathways in the specific strain as well as in other bacterial species, forming three possible pathways (Figure 1). In Pathway 1, degradation occurs via modified ortho-cleavage, where in the first step, there is a ring cleavage in DCA; then, chloro intermediates transform into maleylacetate and finally enter the citric acid cycle. The pathway was described by Bers et al. (2011) based on the study of linuron degradation in *Variovorax* SRS 16 and is supported in part by molecular evidence. Notably, none of the reactions in the modified ortho-cleavage pathway was identified by automatic reconstruction. Alternatively, we also included two hypothetical pathways (not specifically detected for SRS 16), Pathways 2 and 3 (Figure 1), for the degradation of the herbicide hydrolysis product (Arora, 2015; Hussain et al., 2015). Pathway 2 follows the removal of successive dehalogenation and the formation of aniline from DCA. The product aniline enters the ortho-cleavage pathway through catechol to the core metabolism. Pathway 3 begins with the dehalogenation of DCA with the formation of 4-chloroaniline. 4-Chloroaniline is further converted to catechol derivatives, which leads to the meta-cleavage pathway. Some of the enzymes involved in these pathways were detected as part of the automatic reconstruction, and missing reactions were manually added (Figure 1). In the present study, simulations were carried out considering all three pathways, with Pathway 1 only and with Pathways 2 and 3 together; the same results were obtained when considering all the pathways together and with Pathway 1 only. The *Variovorax* sp. SRS 16 strain is documented to grow on linuron alone as it utilizes linuron as a sole carbon and nitrogen source in minimal media (Sørensen and Aamand, 2003). Sørensen et al. (2009) reported a strong enhancing effect of nitrogen (ammonium sulfate) on the degradation rate but not of carbon. These experimental observations were successfully captured by model simulations, indicating a stronger impact of nitrogen supplement (ammonium sulfate) over a carbon supplement (glucose) as enhancers of degradation. Simulation outcomes are consistent while using different pathway alternatives (Figure 2). In accordance with the documented mineralization of linuron (Sørensen et al., 2009), simulations indicate that linuron is converted into central-metabolism metabolites and biomass, where none of the degradation by-products (Figure 1) accumulate in the medium. The predicted production of biomass is fully correlated with linuron degradation (Supplementary File 6), providing additional evidence of full degradation.

### Simulation-Based Predictions for Potential Enhancers of Linuron Degradation

**FIGURE 3:**
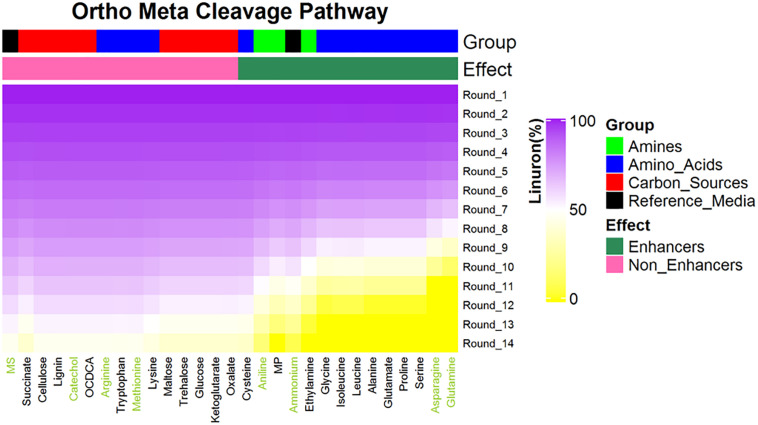
Predictions for linuron degradation by SRS 16 in minimal media supplemented with linuron and 28 compounds (selected carbon and nitrogen sources). Compounds are ranked according the linuron amount in the ninth simulation rounds. All media contain linuron. Reference medium: MS = minimal solution + linuron + no supplement, MP = 2-methyl propanamine. A total of seven metabolites (green font) were selected for experimental validation. Simulations assuming Pathways 2 and 3; simulations assuming alternative pathways are provided in Supplementary File 7.

**FIGURE 4:**
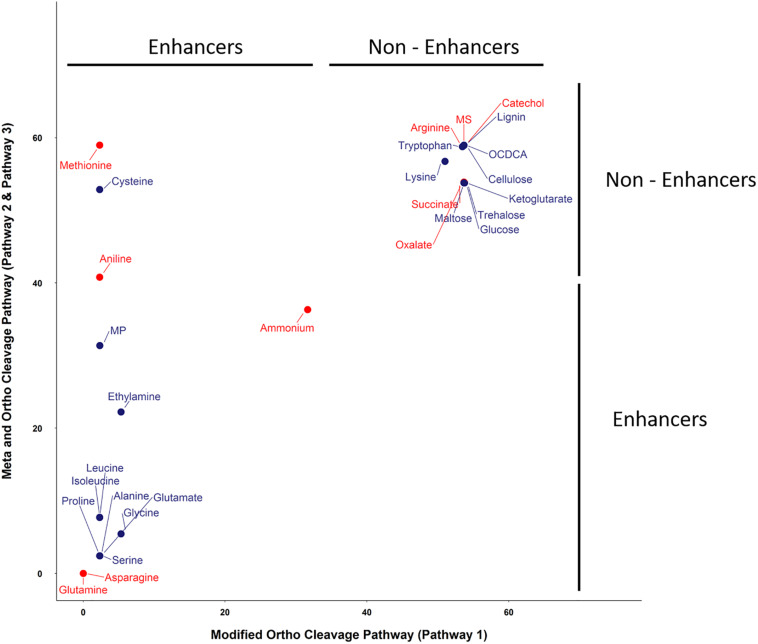
Linuron degradation status (at round 10) under the influence of supplements with the ortho- and meta-cleavage pathway (Pathways 2 and 3) vs. the modified ortho-cleavage pathway (Pathway 1). Substrates in red were selected for further validation in the lab experiment. The x and y scales represent the amount of linuron (%).

The SRS 16 linuron degradation behavior under the influence of specific carbon (succinic acid) and nitrogen (ammonium salt) sources was extensively studied and reported (Sørensen et al., 2009). Here, we used simulations to further explore the effect of media supplements that can serve as potential degradation enhancers. We simulated growth in 120 different media combinations, each supplementing the linuron-containing minimal mineral media with a single exchange metabolite. The list of metabolites, following omission of 20 toxic and other non-relevant substances (considering future application in soil) such as diuron and dipeptides, is provided in Supplementary File 4. The effect of 28 exchange supplements selected across the full scale of degradation efficiency is shown in Figure 3. Selected metabolites were chosen to represent the biochemical diversity of carbon (simple sugars, organic acids, and biopolymers) and nitrogen (mainly amino acids, amine, and ammonium) sources that can act as future biostimulants in terms of regulation, costs, and accessibility. Simulations for all compounds were carried out considering both the SRS 16-specific pathway (Pathway 1, Supplementary File 7) and generic degradation pathways (Pathways 2 and 3, Figure 3). Different supplements were predicted to have variable impact on the growth of *Variovorax* SRS 16 and the degradation rate of linuron. In a reference medium containing linuron only (MS), 40% of the linuron was degraded at the 11th simulation round. The predictions stratify a group of enhancers with variable degrees of linuron degradation in the 11th iteration (>40–100%), in comparison to non-enhancers (40% linuron degradation, as in MS). All the carbon sources and several amino acids (arginine, lysine, methionine, cysteine, and tryptophan) were classified as non-enhancers vs. nitrogen sources that are predicted to expedite degradation at various degrees, with some variations depending on the simulation pathways used. Overall, considering the different pathway options, the rate of degradation broadly follows the same trend where most amino acids act as moderate enhancers, where on the 11th round, 50–75% of linuron is degraded. Fast enhancers include glutamine and asparagine (∼100% degraded in the final, 14th, round in comparison to 60% in MS). Though groups of enhancers vs. non-enhancers are similar when considering both simulation pathways (Figure 3 and Supplementary File 7), differences are observed in internal ranking as presented in Figure 4. The major difference found is that in Pathway 1, methionine and cysteine influence the degradation rate positively, whereas in Pathways 2 and 3, they are categorized as non-enhancers.

### *In vitro* Validation of Potential Enhancers of Linuron Degradation by *Variovorax* sp. SRS 16

**FIGURE 5:**
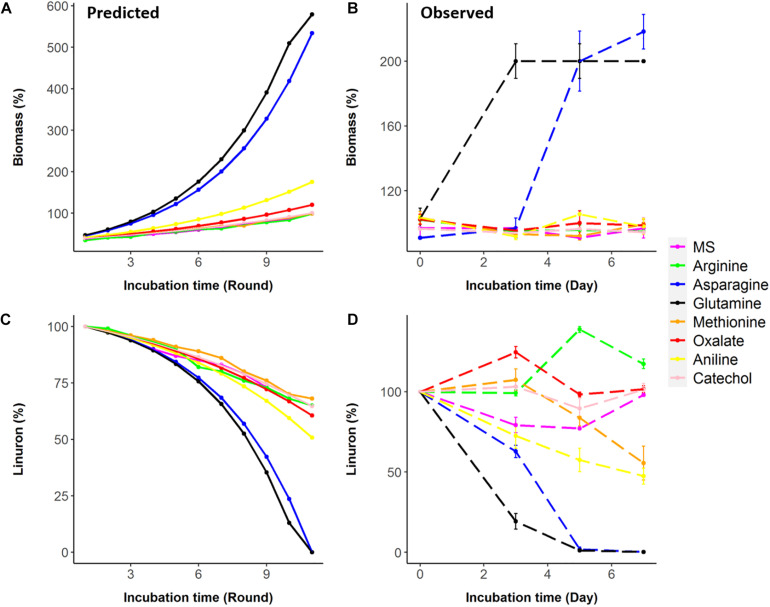
Predictions (based on Pathways 2 and 3) vs. observations for biomass and linuron degradation by *Variovorax* SRS 16. **(A,B)** Biomass predicted and observed. **(C,D)** Linuron degradation predicted and observed.

Seven metabolites representing a range of predicted degradation enhancement potential and biochemical characteristics (Figure 3) were selected for validation. These metabolites include glutamine and asparagine as strong enhancers; aniline as a moderate enhancer; arginine, oxalic acid, and catechol as weak enhancers (Figure 3); and methionine whose enhancement potential depends on the choice of simulation pathway (non-enhancer in Pathway 1 and strong enhancer in Pathways 2 and 3, Figure 4). The simulations (growth and degradation) for the seven selected substrates assuming degradation Pathways 2 and 3 are shown in Figure 5. Simulations considering Pathways 1 and degradation Pathways 1–3 are shown in Supplementary File 8. Overall, the laboratory experiments supported predictions in the majority of the cases. Growth experiments (Figure 5B) are fully consistent with predictions, dividing supplement as non-enhancers vs. enhancers. In agreement with predictions, a slow (negligible) growth rate was found in MS and in MS supplemented by arginine, catechol, aniline, and methionine. A significantly higher growth rate was recorded in the presence of glutamine and asparagine (*p* < 0.05). Degradation results (Figure 5D) are overall consistent with growth results (Figure 5B), with significant stratification of enhancers (glutamine and asparagine) vs non-enhancers (*p* < 0.05). Aniline and methionine had a non-significantly stronger enhancement effect in comparison to MS and were classified into the non-enhancers group (*p* < 0.05 repeated-measures ANOVA), consistent with simulations in Pathways 2 and 3.

The results provide an insight that some supplements (associated with nitrogen metabolism) have a strong enhancing effect that helps in the conversion of linuron into potential cellular building blocks.

## Discussion

Linuron is considered a stable phenyl urea herbicide. Under field conditions, the half-life of linuron varies from 30 to 150 days in different soil types, with an estimated average half-life of 60 days (Swarcewicz et al., 2013). DCA, its primary degradation intermediate, is reported to be more toxic. Therefore, complete mineralization of this compound is desired. *Variovorax* SRS 16, a member of a genus reported as an efficient degrader of phenyl urea herbicide, is widely known for its ability to mineralize linuron (Sørensen et al., 2009). Stimulating the growth of this degrader through adding compounds to a contaminated site is a potential strategy for enhancing its activity while bypassing the need of introducing exogenous microbes. Here, we describe the construction of a genome-scale metabolic model of *Variovorax* sp. strain SRS 16 aiming at finding model-based solutions for accelerating its linuron degradation activity. Construction followed automatic and manual protocols, and iterative simulations were applied for predicting potential compounds that promote the degradation of linuron. Predictions for significant degradation enhancers were confirmed by wet laboratory experiments.

Although, overall, the study demonstrates that model predictions can guide metabolic exploration toward predicting biochemical routes leading to enhanced degradation activity, several limitations of this work should be acknowledged. First, further adaptations are required for the current version of iRZ1425 to meet the requirements of gold standard models (Thiele and Palsson, 2010). Mainly, the biomass composition is an approximation that relies on automatic protocols and genomic information (Oh et al., 2007; Henry et al., 2010), similar to other recent works (Bordel et al., 2019; Naizabekov and Lee, 2020; Ofaim et al., 2020). The construction of a biomass reaction specific to the SRS 16 reaction requires layers of experimental data that are currently missing, including the precursors of general biomass constructions (for example, proteins, lipids, and species-specific components). Collection of currently lacking experimental data will be incorporated in future versions of the model to define an accurate biomass objective function using, for example, the BOFdat computational platform (Lachance et al., 2019). Second, though the current analysis demonstrates the use of model-based solutions for accelerating linuron degradation activity, the mechanism explaining the different effects of the metabolites tested remains unaddressed here. Simulations, supported by experimental evidence from the current study as well as previous reports (Sørensen et al., 2009), clearly point to nitrogenous compounds being advantageous in comparison to carbon ones. However, nitrogen content *per se* is insufficient for predicting the stimulating effect of compounds, and degradation efficiency cannot be ranked according to N content or to the C:N ratio. For example, the efficient stimulants glutamine and asparagine have C:N ratios of 5:2 and 4:2, respectively, in comparison to 6:4 in the non-efficient supplement arginine (Supplementary File 4). Simulation outcomes cannot be predetermined based solely on the C and/or N content of media supplements but rather by considering the complementary stoichiometric effect of multiple chemical reaction cascades, or pathways, that construct the genome-scale metabolic network. Similarly, simulation outcomes cannot be predicted based on compound structural properties, for example, different biostimulation potential of the two peripheral aromatic compounds, aniline and catechol.

Thus, the simulation predictions reflect the complexity of cellular processes and the balance between different metabolic pathways contributing to growth. The simulations provide multiple optimal solutions for the internal fluxes involved in central metabolism and hence are not sufficient for deciphering the mechanism by which environmental (media dependent) conditions affect linuron degradation. However, the integration of simulation data with ‘omics data (e.g., metabolomics, proteomics, and transcriptomics) can reduce the solution space and shed light on the fine-tuning of cellular activity, considering the need to balance between myriads of constraints. The model provides a platform for future sequential studies through the integration of metabolomic and transcriptomic data (similar to Hadadi et al., 2020), allowing the exploration of the mechanism of linuron degradation as well as other aspects of *Variovorax* sp. strain SRS 16 biochemistry. Such additional experiments are required in order to complement model predictions regarding the mechanism behind accelerating the phenomenon. For example, transcriptomics/proteomics studies targeting the effect of different media supplements (e.g., amino acids) would be beneficial to track the molecular processes considering different degradation scenarios.

Though current analysis requires additional layers on information in order to provide a description of the degradation mechanism, the analysis provides several insights that can be further explored in future studies. Model construction suggests three alternative routes for the degradation of linuron. Pathway 1 (modified ortho-cleavage pathway) relies on a detailed description of linuron degradation at strain level (Öztürk et al., 2020). *Variovorax* SRS 16 was reported to contain the gene coding for the amidase *LibA*, which hydrolyzes linuron in *N*,*O*-dimethyl hydroxylamine and DCA. The catabolic pathway for DCA degradation into components of central metabolism is suggested to involve conversion of 4,5-dichlorocatechol to oxoadipate (modified ortho-cleavage pathway) (Bers et al., 2011). Alternative degradation pathways of DCA were reported in other bacterial genera (Dejonghe et al., 2002; Breugelmans et al., 2010). In the current study, automatic construction based on the SRS 16 genome sequence suggested the partial presence of enzymes in chloroaniline or chlorocatechol metabolism (Pathways 2 and 3), where enzymes from the modified ortho-cleavage pathway (Pathway 1) were not detected. The missing/partial degradation pathways were completed through a gap-filling procedure (Figure 1). Here, the possibility of the presence of the ortho-cleavage pathway and meta-cleavage pathway along with the existing modified ortho-cleavage pathway is proposed based on genome data. The presence of partial Pathways 2 and 3 indicates that SRS 16 might be using these pathways for linuron mineralization under different conditions. Simulations in the SRS 16 model with complete Pathways 2 and 3 provided similar results to those obtained with Pathway 1 for most of the supplements (such as glutamine, asparagine, and aniline), with some exceptions such as methionine.

In the present study, it is shown that reconstruction is important for the study of bacterial physiology, allowing the simulation of degradation outcomes considering multiple conditions. It is well known that the degradation process is influenced by various physicochemical and nutritional factors (Kanissery and Sims, 2011). The availability of carbon and nitrogen sources (nutrients) significantly affects the efficiency of degradation (Yassir et al., 1998; Wu et al., 2011). Here, simulations suggest the importance of specific supplements to increase the degradation rate. Simulations predicted variations in the enhancement potential of different supplements, further supported by experimental validation. As a single cell is a complex network of reactions and metabolites, supplements can have a variable effect associated with the activation of different sets of reactions under each media condition. Integration of model predictions with omics data is useful for revealing the metabolic shifts in the microbial cell (Alam et al., 2010). To date, several strategies have been adopted in order to accelerate the biodegradation of herbicides in the environment, ranging from optimizing media to constructing transgenics (Azab et al., 2018; Li et al., 2020). Genome-scale modeling approaches along with omics technology have established their importance in biotechnology and medicine (Zhang and Hua, 2016). They are continuously appreciated for their role in the degradation of pollutants (Faust, 2019; Xu et al., 2019; Cardozo et al., 2020) and in other environmental problems related to agricultural soils (Mazzola and Freilich, 2017). Recently, Ofaim et al. (2020) demonstrated the use of genome-scale modeling for optimizing atrazine degradation by *P. aurescens* TC1. The current study demonstrated that genome-scale modeling allows the optimization of microbial activity, leading to herbicide biodegradation. The integration of predictive biology helped to screen the effect of the higher number of supplements on the degradation by SRS 16. Such supplement-derived amendment might reflect exchanges in the indigenous community where the degrader can be supported by non-degrader species (Xu et al., 2019) and accelerate the degradation rate.

In this way, computational biology can reduce cost, time, and effort (mainly related to gene manipulation) and save resources to identify the optimal solutions for a specific phenomenon. The association of genome-scale metabolic models with the omics approach can help to unravel the hidden facts of metabolism (Massaiu et al., 2019). Thus, there is a strong possibility of improvement in degradation processes through the contribution of modeling as an environmental biotechnology approach.

## Data Availability Statement

The original contributions presented in the study are included in the article/Supplementary Materials, further inquiries can be directed to the corresponding author.

## Author Contributions

KD, RZ, ZR, and SF designed the study and drafted the manuscript. RZ constructed the metabolic model. KD participated in model construction, led the design and analysis of computational and experimental results, and wrote the manuscript. SM participated in computational data analysis. NK and DB performed the wet laboratory experiments. BÖ, RA, and HE analyzed and reviewed the results. All authors carried out writing and improvement of manuscript.

## Conflict of Interest

BÖ was employed by the Leibniz Institute DSMZ, German Collection of Microorganisms and Cell Cultures, Braunschweig, Germany. The remaining authors declare that the research was conducted in the absence of any commercial or financial relationships that could be construed as a potential conflict of interest.

## References

[B1] Alam M. T. Merlo M. E. Hodgson D. A. Wellington E. M. H. Takano E. Breitling R. (2010). Metabolic modeling and analysis of the metabolic switch in *Streptomyces coelicolor*. *BMC Genom.* 11:202. 10.1186/1471-2164-11-202 20338070 PMC2853524

[B2] Arora P. K. (2015). Bacterial degradation of monocyclic aromatic amine. *Front. Microbiol.* 6:820. 10.3389/fmicb.2015.00820 26347719 PMC4539516

[B3] Azab E. Kebeish R. Hegazy A. K. (2018). Expression of the human gene CYP1A2 enhances tolerance and detoxification of the phenylurea herbicide linuron in *Arabidopsis thaliana* plants and *Escherichia coli*. *Environ. Pollut.* 238 281–290. 10.1016/j.envpol.2018.03.025 29573710

[B4] Bao H. Wang J. Zhang H. Li J. Li H. Wu F. (2020). Effects of biochar and organic substrates on biodegradation of polycyclic aromatic hydrocarbons and microbial community structure in PAHs-contaminated soils. *J. Hazard. Mater.* 385:121595. 10.1016/j.jhazmat.2019.121595 31744730

[B5] Bers K. Leroy B. Breugelmans P. Albers P. Lavigne R. Sørensen S. R. (2011). A novel hydrolase identified by genomic-proteomic analysis of phenylurea herbicide mineralization by *Variovorax* sp. strain SRS16. *Appl. Environ. Microbiol.* 77 8754–8764. 10.1128/AEM.06162-11 22003008 PMC3233098

[B6] Bhat S. A. Qadri H. Cui G. Li F. (2020). “Remediation of pesticides through microbial and phytoremediation techniques,” in *Fresh Water Pollution Dynamics and Remediation*, eds Qadri H. Bhat R. Mehmood M. Dar G. (Singapore: Springer), 235–245. 10.1007/978-981-13-8277-2_13

[B7] Bordel S. Rojas A. Muñoz R. (2019). Reconstruction of a genome scale metabolic model of the polyhydroxybutyrate producing methanotroph *Methylocystis parvus* OBBP. *Microb. Cell Fact.* 18 1–11. 10.1186/s12934-019-1154-5 31170985 PMC6554988

[B8] Breugelmans P. Leroy B. Bers K. Dejonghe W. Wattiez R. De Mot R. (2010). Proteomic study of linuron and 3,4-dichloroaniline degradation by *Variovorax* sp. WDL1: evidence for the involvement of an aniline dioxygenase-related multicomponent protein. *Res. Microbiol.* 161 208–218. 10.1016/j.resmic.2010.01.010 20146937

[B9] Calmels C. McCann A. Malphettes L. Andersen M. R. (2019). Application of a curated genome-scale metabolic model of CHO DG44 to an industrial fed-batch process. *Metab. Eng.* 51 9–19. 10.1016/j.ymben.2018.09.009 30227251

[B10] Cardozo M. De Almeida J. S. F. D. De A. Cavalcante S. F. Salgado J. R. S. Gonçalves A. S. (2020). Biodegradation of organophosphorus compounds predicted by enzymatic process using molecular modelling and observed in soil samples through analytical techniques and microbiological analysis: a comparison. *Molecules* 25:58. 10.3390/molecules25010058 31878010 PMC6982719

[B11] Chen I. M. A. Chu K. Palaniappan K. Pillay M. Ratner A. Huang J. (2019). IMG/M v.5.0: an integrated data management and comparative analysis system for microbial genomes and microbiomes. *Nucleic Acids Res.* 47 D666–D677. 10.1093/nar/gky901 30289528 PMC6323987

[B12] Covert M. W. Schilling C. H. Famili I. Edwards J. S. Goryanin I. I. Selkov E. (2001). Metabolic modeling of microbial strains in silico. *Trends Biochem. Sci.* 26 179–186. 10.1016/S0968-0004(00)01754-0 11246024

[B13] de Souza R. M. Seibert D. Quesada H. B. de Jesus Bassetti F. Fagundes-Klen M. R. Bergamasco R. (2020). Occurrence, impacts and general aspects of pesticides in surface water: a review. *Process Saf. Envrion.* 135 22–37. 10.1016/j.psep.2019.12.035

[B14] Dejonghe W. Berteloot E. Goris J. Boon N. Crul K. Maertens S. (2003). Synergistic degradation of linuron by a bacterial consortium and isolation of a single linuron-degrading *Variovorax* strain. *Appl. Environ. Microbiol.* 69 1532–1541. 10.1128/AEM.69.3.1532-1541.2003 12620840 PMC150106

[B15] Dejonghe W. Goris J. Dierickx A. De Dobbeleer V. Crul K. De Vos P. (2002). Diversity of 3-chloroaniline and 3,4-dichloroaniline degrading bacteria isolated from three different soils and involvement of their plasmids in chloroaniline degradation. *FEMS Microbiol. Ecol.* 42 315–325. 10.1016/S0168-6496(02)00344-6 19709291

[B16] Devoid S. Overbeek R. DeJongh M. Vonstein V. Best A. A. Henry C. (2013). Automated genome annotation and metabolic model reconstruction in the SEED and model SEED. *Methods Mol. Biol.* 985 17–45. 10.1007/978-1-62703-299-5_2 23417797

[B17] Faria J. P. Rocha M. Rocha I. Henry C. S. (2018). Methods for automated genome-scale metabolic model reconstruction. *Biochem. Soc. Trans.* 46 931–936. 10.1042/BST20170246 30065105

[B18] Faust K. (2019). Microbial consortium design benefits from metabolic modeling. *Trends Biotechnol.* 37 123–125. 10.1016/j.tibtech.2018.11.004 30477738

[B19] Feist A. M. Zielinski D. C. Orth J. D. Schellenberger J. Herrgard M. J. Palsson B. O. (2010). Model-driven evaluation of the production potential for growth-coupled products of *Escherichia coli*. *Metab. Eng.* 12 173–186. 10.1016/j.ymben.2009.10.003 19840862 PMC3125152

[B20] Garcês A. Pires I. Rodrigues P. (2020). Teratological effects of pesticides in vertebrates: a review. *J. Environ. Sci. Heal. Part B Pestic. Food Contam. Agric. Wastes* 55 75–89. 10.1080/03601234.2019.1660562 31516070

[B21] García-Jiménez B. De La Rosa T. Wilkinson M. D. (2018). MDPbiome: microbiome engineering through prescriptive perturbations. *Bioinformatics* 34 i838–i847. 10.1093/bioinformatics/bty562 30423107 PMC6129268

[B22] Gu C. Kim G. B. Kim W. J. Kim H. U. Lee S. Y. (2019). Current status and applications of genome-scale metabolic models. *Genome Biol.* 20 1–18. 10.1186/s13059-019-1730-3 31196170 PMC6567666

[B23] Hadadi N. Pandey V. Chiappino-Pepe A. Morales M. Gallart-Ayala H. Mehl F. (2020). Mechanistic insights into bacterial metabolic reprogramming from omics-integrated genome-scale models. *NPJ Syst. Biol. Appl.* 6:1. 10.1038/s41540-019-0121-4 32001719 PMC6946695

[B24] Hao L. Wang Y. Wang C. Wu Q. Wang Z. (2019). A magnetic covalent aromatic polymer as an efficient and recyclable adsorbent for phenylurea herbicides. *Microchim. Acta* 186:431. 10.1007/s00604-019-3583-6 31187290

[B25] Hasanuzzaman M. Mohsin S. M. Bhuyan M. H. M. B. Bhuiyan T. F. Anee T. I. Masud A. A. C. (2020). “Phytotoxicity, environmental and health hazards of herbicides: challenges and ways forward,” in *Agrochemicals, Detection, Treatement and Remediation*, ed Vara Prasad M. N. (Oxford: Butterworth-Heinemann), 55–99. 10.1016/B978-0-08-103017-2.00003-9

[B26] Henry C. S. Dejongh M. Best A. A. Frybarger P. M. Linsay B. Stevens R. L. (2010). High-throughput generation, optimization and analysis of genome-scale metabolic models. *Nat. Biotechnol.* 28 977–982. 10.1038/nbt.1672 20802497

[B27] Horemans B. Bers K. Romero E. R. Juan E. P. Dunon V. De Mot R. (2016). Functional redundancy of linuron degradation in microbial communities in agricultural soil and biopurification systems. *Appl. Environ. Microbiol.* 82 2843–2853. 10.1128/AEM.04018-15 26944844 PMC4836412

[B28] Hucka M. Finney A. Sauro H. M. Bolouri H. Doyle J. C. Kitano H. (2003). The systems biology markup language (SBML): a medium for representation and exchange of biochemical network models. *Bioinformatics* 19 524–531. 10.1093/bioinformatics/btg015 12611808

[B29] Hussain S. Arshad M. Springael D. Sørensen S. R. Bending G. D. Devers-Lamrani M. (2015). Abiotic and biotic processes governing the fate of Phenylurea herbicides in soils: a review. *Crit. Rev. Environ. Sci. Technol.* 45 1947–1998. 10.1080/10643389.2014.1001141

[B30] Kanehisa M. Sato Y. Kawashima M. Furumichi M. Tanabe M. (2016). KEGG as a reference resource for gene and protein annotation. *Nucleic Acids Res.* 44 D457–D462. 10.1093/nar/gkv1070 26476454 PMC4702792

[B31] Kanissery R. G. Sims G. K. (2011). Biostimulation for the enhanced degradation of herbicides in soil. *Appl. Environ. Soil Sci.* 2011 843450. 10.1155/2011/843450

[B32] Karp P. D. (2002). The MetaCyc database. *Nucleic Acids Res.* 30 59–61. 10.1093/nar/30.1.59 11752254 PMC99148

[B33] Katsumata H. Kobayashi T. Kaneco S. Suzuki T. Ohta K. (2011). Degradation of linuron by ultrasound combined with photo-Fenton treatment. *Chem. Eng. J.* 166 468–473. 10.1016/j.cej.2010.10.073

[B34] Kovács K. Farkas J. Veréb G. Arany E. Simon G. Schrantz K. (2016). Comparison of various advanced oxidation processes for the degradation of phenylurea herbicides. *J. Environ. Sci. Heal. Part B Pestic. Food Contam. Agric. Wastes* 51 205–214. 10.1080/03601234.2015.1120597 26764571

[B35] Lachance J. C. Lloyd C. J. Monk J. M. Yang L. Sastry A. V. Seif Y. (2019). BOFDAT: generating biomass objective functions for genome-scale metabolic models from experimental data. *PLoS Comput. Biol.* 15:e1006971. 10.1371/journal.pcbi.1006971 31009451 PMC6497307

[B36] Lerner H. Öztürk B. Dohrmann A. B. Thomas J. Marchal K. De Mot R. (2020). Culture-independent analysis of Linuron-mineralizing Microbiota and functions in on-Farm biopurification systems via DNA-Stable isotope probing: comparison with enrichment culture. *Environ. Sci. Technol.* 54 9387–9397. 10.1021/acs.est.0c02124 32569463

[B37] Li C. Zhang X. Lu Y. Fan Z. Wang T. Zhang G. (2020). Cometabolic degradation of p-chloroaniline by the genus *Brevibacillus* bacteria with extra carbon sources. *J. Hazard. Mater.* 383:121198. 10.1016/j.jhazmat.2019.121198 31541955

[B38] Liu J. (2010). *Phenylurea Herbicides*, 3rd Edn, Amsterdam: Elsevier Inc, 10.1016/B978-0-12-374367-1.00080-X

[B39] Mahadevan R. Schilling C. H. (2003). The effects of alternate optimal solutions in constraint-based genome-scale metabolic models. *Metab. Eng.* 5 264–276. 10.1016/j.ymben.2003.09.002 14642354

[B40] Massaiu I. Pasotti L. Sonnenschein N. Rama E. Cavaletti M. Magni P. (2019). Integration of enzymatic data in *Bacillus subtilis* genome-scale metabolic model improves phenotype predictions and enables in silico design of poly-γ-glutamic acid production strains. *Microb. Cell Fact* 18 1–20. 10.1186/s12934-018-1052-2 30626384 PMC6325765

[B41] Mazzola M. Freilich S. (2017). Prospects for biological soilborne disease control: application of indigenous versus synthetic microbiomes. *Phytopathology* 107 256–263. 10.1094/PHYTO-09-16-0330-RVW 27898265

[B42] Monk J. M. Lloyd C. J. Brunk E. Mih N. Sastry A. King Z. (2017). iML1515, a knowledgebase that computes *Escherichia coli* traits. *Nat. Biotechnol.* 35 904–908. 10.1038/nbt.3956 29020004 PMC6521705

[B43] Naizabekov S. Lee E. Y. (2020). Genome-scale metabolic model reconstruction and in silico investigations of methane metabolism in *Methylosinus trichosporium* ob3b. *Microorganisms* 8:437. 10.3390/microorganisms8030437 32244934 PMC7144005

[B44] Norsigian C. J. Pusarla N. McConn J. L. Yurkovich J. T. Dräger A. Palsson B. O. (2020). BiGG Models 2020: multi-strain genome-scale models and expansion across the phylogenetic tree. *Nucleic Acids Res.* 48 D402–D406. 10.1093/nar/gkz1054 31696234 PMC7145653

[B45] Ofaim S. Zarecki R. Porob S. Gat D. Lahav T. Kashi Y. (2020). Genome-scale reconstruction of *Paenarthrobacter aurescens* TC1 metabolic model towards the study of atrazine bioremediation. *Sci. Rep.* 10:13019. 10.1038/s41598-020-69509-7 32747737 PMC7398907

[B46] Oh Y. K. Palsson B. O. Park S. M. Schilling C. H. Mahadevan R. (2007). Genome-scale reconstruction of metabolic network in *Bacillus subtilis* based on high-throughput phenotyping and gene essentiality data. *J. Biol. Chem.* 282 28791–28799. 10.1074/jbc.M703759200 17573341

[B47] Orth J. D. Conrad T. M. Na J. Lerman J. A. Nam H. Feist A. M. (2011). A comprehensive genome-scale reconstruction of *Escherichia coli* metabolism-2011. *Mol. Syst. Biol.* 7:535. 10.1038/msb.2011.65 21988831 PMC3261703

[B48] Overbeek R. Olson R. Pusch G. D. Olsen G. J. Davis J. J. Disz T. (2014). The SEED and the rapid annotation of microbial genomes using subsystems technology (RAST). *Nucleic Acids Res.* 42 206–214. 10.1093/nar/gkt1226 24293654 PMC3965101

[B49] Öztürk B. Werner J. Meier-Kolthoff J. P. Bunk B. Spröer C. Springael D. (2020). Comparative genomics suggests mechanisms of genetic adaptation toward the catabolism of the phenylurea herbicide linuron in *Variovorax*. *Genome Biol. Evol.* 12 827–841. 10.1093/gbe/evaa085 32359160 PMC7313664

[B50] Park J. M. Kim T. Y. Lee S. Y. (2011). Genome-scale reconstruction and in silico analysis of the *Ralstonia eutropha* H16 for polyhydroxyalkanoate synthesis, lithoautotrophic growth, and 2-methyl citric acid production. *BMC Syst. Biol.* 5:101. 10.1186/1752-0509-5-101 21711532 PMC3154180

[B51] Pimviriyakul P. Wongnate T. Tinikul R. Chaiyen P. (2020). Microbial degradation of halogenated aromatics: molecular mechanisms and enzymatic reactions. *Microb. Biotechnol.* 13 67–86. 10.1111/1751-7915.13488 31565852 PMC6922536

[B52] Raman K. Chandra N. (2009). Flux balance analysis of biological systems: applications and challenges. *Brief. Bioinform.* 10 435–449. 10.1093/bib/bbp011 19287049

[B53] Rana P. Berry C. Ghosh P. Fong S. S. (2020). Recent advances on constraint-based models by integrating machine learning. *Curr. Opin. Biotechnol.* 64 85–91. 10.1016/j.copbio.2019.11.007 31812921

[B54] Reddy P. V. L. Kim K. H. (2015). A review of photochemical approaches for the treatment of a wide range of pesticides. *J. Hazard. Mater.* 285 325–335. 10.1016/j.jhazmat.2014.11.036 25528231

[B55] Risso C. Sun J. Zhuang K. Mahadevan R. DeBoy R. Ismail W. (2009). Genome-scale comparison and constraint-based metabolic reconstruction of the facultative anaerobic Fe(III)-reducer *Rhodoferax ferrireducens*. *BMC Genom.* 10:447. 10.1186/1471-2164-10-447 19772637 PMC2755013

[B56] Scheibe T. D. Mahadevan R. Fang Y. Garg S. Long P. E. Lovley D. R. (2009). Coupling a genome-scale metabolic model with a reactive transport model to describe in situ uranium bioremediation. *Microb. Biotechnol.* 2 274–286. 10.1111/j.1751-7915.2009.00087.x 21261921 PMC3815847

[B57] Sørensen S. R. Aamand J. (2003). Rapid mineralisation of the herbicide isoproturon in soil from a previously treated Danish agricultural field. *Pest Manag. Sci.* 59 1118–1124. 10.1002/ps.739 14561069

[B58] Sørensen S. R. Rasmussen J. Jacobsen C. S. Jacobsen O. S. Juhler R. K. Aamand J. (2005). Elucidating the key member of a linuron-mineralizing bacterial community by PCR and reverse transcription-PCR denaturing gradient gel electrophoresis 16S rRNA gene fingerprinting and cultivation. *Appl. Environ. Microbiol.* 71 4144–4148. 10.1128/AEM.71.7.4144-4148.2005 16000836 PMC1169018

[B59] Sørensen S. R. Simonsen A. Aamand J. (2009). Constitutive mineralization of low concentrations of the herbicide linuron by a *Variovorax* sp. strain. *FEMS Microbiol. Lett.* 292 291–296. 10.1111/j.1574-6968.2009.01501.x 19187207

[B60] Swarcewicz M. Gregorczyk A. Sobczak J. (2013). Comparison of linuron degradation in the presence of pesticide mixtures in soil under laboratory conditions. *Environ. Monit. Assess.* 185 8109–8114. 10.1007/s10661-013-3158-7 23525775 PMC3759734

[B61] The UniProt Consortium (2019). UniProt: a worldwide hub of protein knowledge The UniProt Consortium. *Nucleic Acids Res.* 47 D506–D515. 10.1093/nar/gky1049 30395287 PMC6323992

[B62] Thiele I. Palsson B. (2010). A protocol for generating a high-quality genome-scale metabolic reconstruction. *Nat. Protoc.* 5 93–121. 10.1038/nprot.2009.203 20057383 PMC3125167

[B63] Turnbull G. A. Ousley M. Walker A. Shaw E. Morgan J. A. W. (2001). Degradation of substituted phenylurea herbicides by *Arthrobacter globiformis* strain D47 and characterization of a plasmid-associated hydrolase Gene, puhA. *Appl. Environ. Microbiol.* 67 2270–2275. 10.1128/AEM.67.5.2270-2275.2001 11319111 PMC92866

[B64] Wang C. Deng Z. L. Xie Z. M. Chu X. Y. Chang J. W. Kong D. X. (2015). Construction of a genome-scale metabolic network of the plant pathogen *Pectobacterium carotovorum* provides new strategies for bactericide discovery. *FEBS Lett.* 589 285–294. 10.1016/j.febslet.2014.12.010 25535697

[B65] Wu R. R. Dang Z. Yi X. Y. Yang C. Lu G. N. Guo C. L. (2011). The effects of nutrient amendment on biodegradation and cytochrome P450 activity of an n-alkane degrading strain of *Burkholderia* sp. GS3C. *J. Hazard. Mater.* 186 978–983. 10.1016/j.jhazmat.2010.11.095 21167642

[B66] Xu X. Zarecki R. Medina S. Ofaim S. Liu X. Chen C. (2019). Modeling microbial communities from atrazine contaminated soils promotes the development of biostimulation solutions. *ISME J.* 13 494–508. 10.1038/s41396-018-0288-5 30291327 PMC6331595

[B67] Yassir A. Rieu C. Soulas G. (1998). Microbial N-dealkylation of Atrazine: effect of exogeneous organic substrates and behaviour of the soil microflora. *Pestic. Sci.* 54 75–82.

[B68] Zhang C. Hua Q. (2016). Applications of genome-scale metabolic models in biotechnology and systems medicine. *Front. Physiol.* 6:413. 10.3389/fphys.2015.00413 26779040 PMC4703781

[B69] Zhang L. Hu Q. Liu B. Li F. Jiang J. D. (2020). Characterization of a Linuron-specific amidohydrolase from the newly isolated bacterium *Sphingobium* sp. Strain SMB. *J. Agric. Food Chem.* 68 4335–4345. 10.1021/acs.jafc.0c00597 32207940

[B70] Zhu Y. Czauderna T. Zhao J. Klapperstueck M. Maifiah M. H. M. Han M. L. (2018). Genome-scale metabolic modeling of responses to polymyxins in *Pseudomonas aeruginosa*. *Gigascience* 7:giy021. 10.1093/gigascience/giy021 29688451 PMC6333913

