# Supplementary material for: Strategies for Enhancing in vitro Degradation of Linuron by Variovorax sp. Strain SRS 16 Under the Guidance of Metabolic Modeling
Source: Front Bioeng Biotechnol. 2021 Apr 15;9:602464. doi: 10.3389/fbioe.2021.602464 (PMC8084104; doi:10.3389/fbioe.2021.602464)
Supplement: Supplementary file 3 [file Table_3.DOCX]

**Strategies for enhancing *in-vitro* degradation of linuron by *Variovorax* sp. strain SRS 16 under the guidance of metabolic modeling**

Kusum Dhakar^1,2¥^, Raphy Zarecki^1,2¥^, Daniella van Bommel^3^, Nadav Knossow^2^, Shlomit Medina^1^, Basak Öztürk^4^, Radi Aly^1^, Hanan Eizenberg^1^, Zeev Ronen^2 ϯ^ & Shiri Freilich^1^*^ϯ^

^1^Newe Ya'ar Research Center, Agricultural Research Organization, Ramat Yishay, Israel, ^2^Department of Environmental Hydrology & Microbiology, Zuckerberg Institute for Water Research, Jacob Blaustein Institutes for Desert Research, Ben-Gurion University of the Negev, Midreshet Ben-Gurion, Israel,

^3^Albert Katz School for Desert Studies Jacob Blaustein Institutes for Desert Research, Ben-Gurion University of the Negev, Midreshet Ben-Gurion, Israel,

^4^Junior Research Group Microbial Biotechnology, Leibniz Institute DSMZ, German Collection of Microorganisms and Cell Cultures, Braunschweig, Germany

Supplementary file_3:

Minimal mineral solution for linuron degradation experiments (Sørensen and Aamand, 2003)

| S.No. | Compound | Quantity (l^-1^) |
| --- | --- | --- |
| 1 | KH2PO4 | 1.36 g |
| 2 | Na2HPO4·2H2O | 1.78 g |
| 3 | MgSO4·7H2O | 0.05 g |
| 4 | CaCl2 | 0.01 g |
| 5 | H3BO4 | 2.86 mg |
| 6 | MnSO4·H2O | 1.54 mg |
| 7 | CuSO4·5H2O | 0.04 mg |
| 8 | ZnCl2 | 0.021 mg |
| 9 | CoCl2·6H2O | 0.041 mg |
| 10 | Na2MoO4·2H2O | 0.025 mg |
| - pH adjusted to 7.2 by using NaOH of 1.0M - Medium was sterilized with standard procedures (121℃ for 20 min) - 1.0 ml of FeCl3·6H2O filter sterilized solution (5.14mg l^-1^) was added to the autoclaved medium | | |
