# Supplementary material for: Strategies for Enhancing in vitro Degradation of Linuron by Variovorax sp. Strain SRS 16 Under the Guidance of Metabolic Modeling
Source: Front Bioeng Biotechnol. 2021 Apr 15;9:602464. doi: 10.3389/fbioe.2021.602464 (PMC8084104; doi:10.3389/fbioe.2021.602464)
Supplement: Supplementary file 6 [file Table_6.DOCX]

**Strategies for enhancing *in-vitro* degradation of linuron by *Variovorax* sp. strain SRS 16 under the guidance of metabolic modeling**

Kusum Dhakar^1,2¥^, Raphy Zarecki^1,2¥^, Daniella van Bommel^3^, Nadav Knossow^2^, Shlomit Medina^1^, Basak Öztürk^4^, Radi Aly^1^, Hanan Eizenberg^1^, Zeev Ronen^2 ϯ^ & Shiri Freilich^1^*^ϯ^

^1^Newe Ya'ar Research Center, Agricultural Research Organization, Ramat Yishay, Israel, ^2^Department of Environmental Hydrology & Microbiology, Zuckerberg Institute for Water Research, Jacob Blaustein Institutes for Desert Research, Ben-Gurion University of the Negev, Midreshet Ben-Gurion, Israel,

^3^Albert Katz School for Desert Studies Jacob Blaustein Institutes for Desert Research, Ben-Gurion University of the Negev, Midreshet Ben-Gurion, Israel,

^4^Junior Research Group Microbial Biotechnology, Leibniz Institute DSMZ, German Collection of Microorganisms and Cell Cultures, Braunschweig, Germany


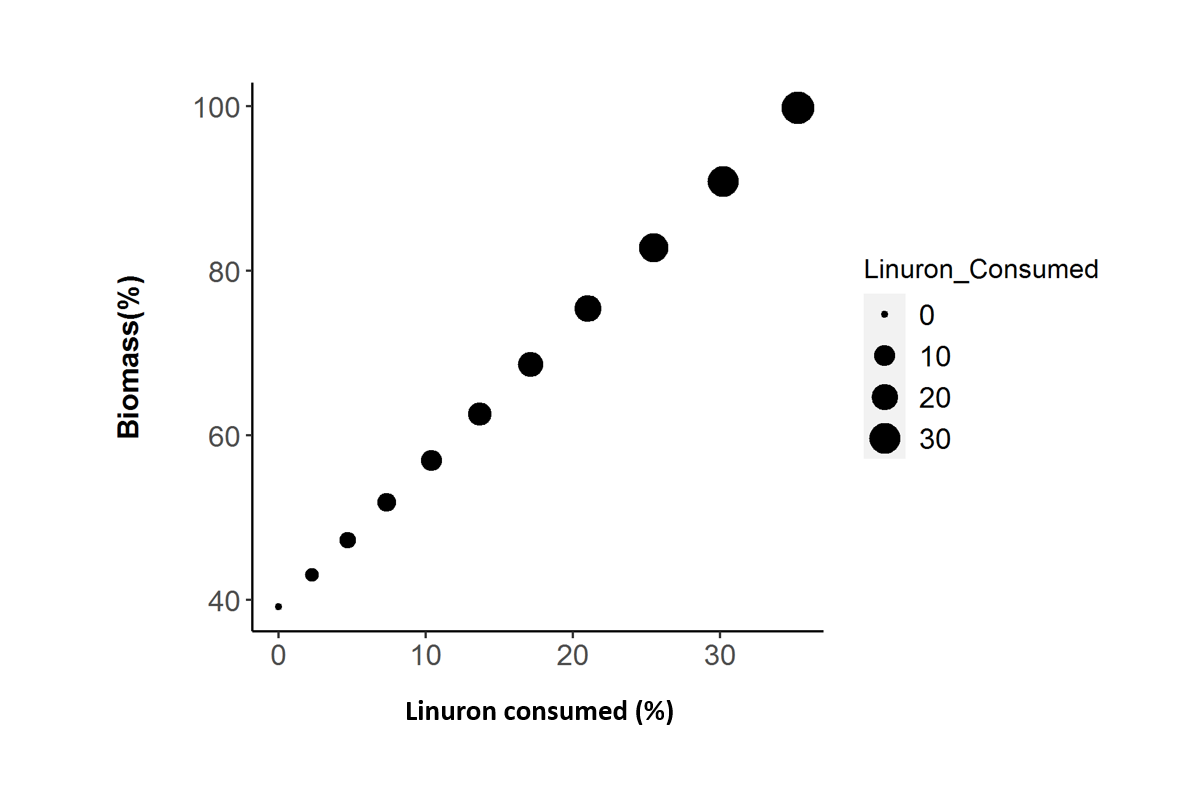
Supplementary file 6. Simulated growth of *Variovorax* sp. SRS16 model in the minimal media supplemented with linuron as sole carbon and nitrogen source
