# Supplementary material for: Strategies for Enhancing in vitro Degradation of Linuron by Variovorax sp. Strain SRS 16 Under the Guidance of Metabolic Modeling
Source: Front Bioeng Biotechnol. 2021 Apr 15;9:602464. doi: 10.3389/fbioe.2021.602464 (PMC8084104; doi:10.3389/fbioe.2021.602464)
Supplement: Supplementary file 7 [file Table_7.DOCX]

**Strategies for enhancing *in-vitro* degradation of linuron by *Variovorax* sp. strain SRS 16 under the guidance of metabolic modeling**

Kusum Dhakar^1,2¥^, Raphy Zarecki^1,2¥^, Daniella van Bommel^3^, Nadav Knossow^2^, Shlomit Medina^1^, Basak Öztürk^4^, Radi Aly^1^, Hanan Eizenberg^1^, Zeev Ronen^2 ϯ^ & Shiri Freilich^1^*^ϯ^

^1^Newe Ya'ar Research Center, Agricultural Research Organization, Ramat Yishay, Israel, ^2^Department of Environmental Hydrology & Microbiology, Zuckerberg Institute for Water Research, Jacob Blaustein Institutes for Desert Research, Ben-Gurion University of the Negev, Midreshet Ben-Gurion, Israel,

^3^Albert Katz School for Desert Studies Jacob Blaustein Institutes for Desert Research, Ben-Gurion University of the Negev, Midreshet Ben-Gurion, Israel,

^4^Junior Research Group Microbial Biotechnology, Leibniz Institute DSMZ, German Collection of Microorganisms and Cell Cultures, Braunschweig, Germany

Supplementary file_7


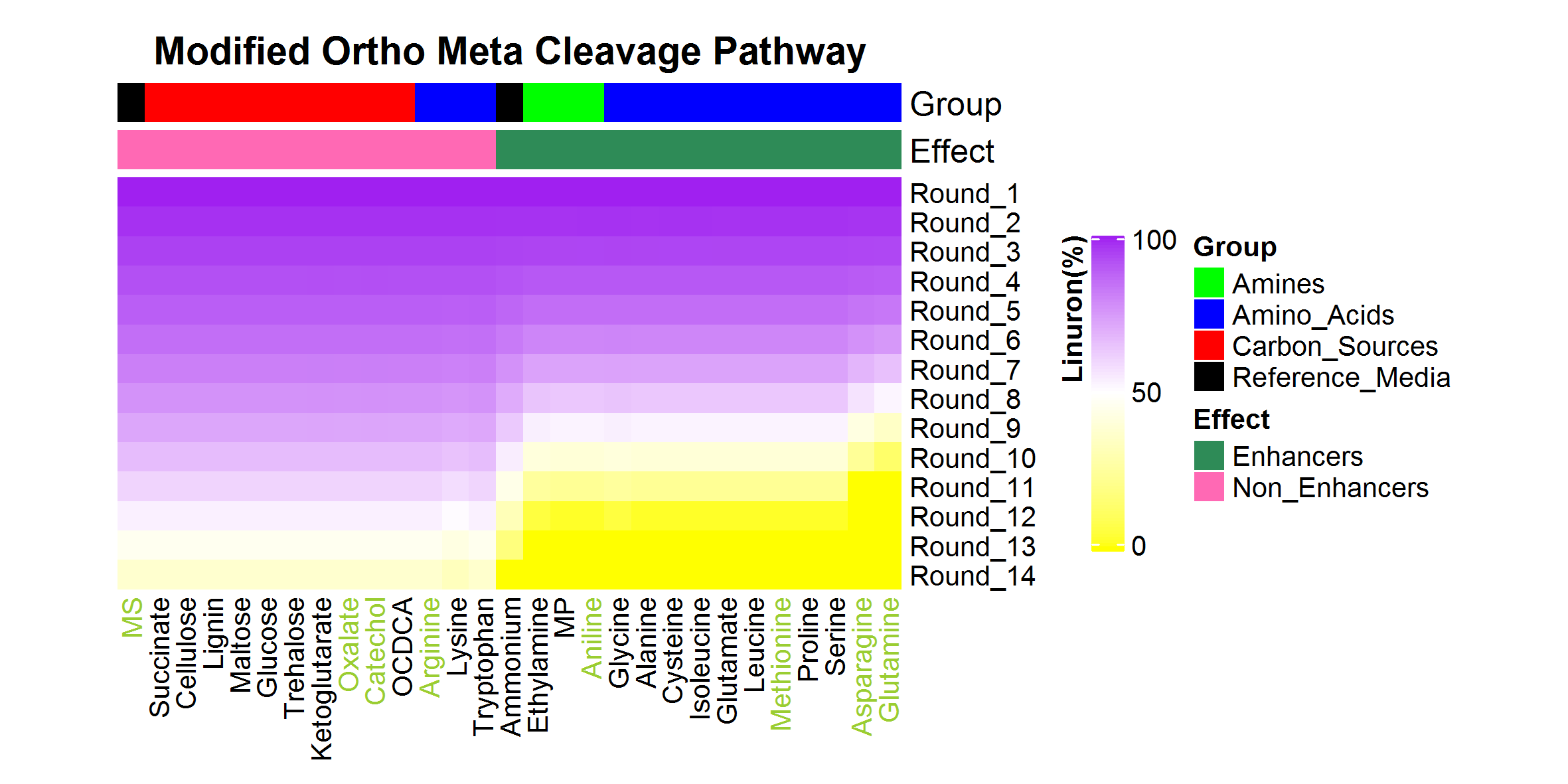


Predictions for linuron degradation by SRS 16 in minimal media supplemented with linuron and 28 compounds (selected carbon and nitrogenous sources). Compounds are ranked according the linuron amount in the 9^th^ simulation rounds. All media contain linuron. Reference medium: MS = Minimal ­­solution + no supplement, MP = 2-methyl propanamine. Color gradient (purple to yellow) represent the amount of linuron (100 to 0 %, respectively). Total 7 metabolites (green) were selected for validation through laboratory experiment. Simulations assume Pathway 1. The simulations are similar with all the pathways together (Pathway 1, 2, &3)
